# Polypharmacy: a challenge for the primary health care of the Brazilian Unified Health System

**DOI:** 10.11606/S1518-8787.2017051007136

**Published:** 2017-09-22

**Authors:** Renata Cristina Rezende Macedo do Nascimento, Juliana Álvares, Augusto Afonso Guerra, Isabel Cristina Gomes, Micheline Rosa Silveira, Ediná Alves Costa, Silvana Nair Leite, Karen Sarmento Costa, Orlando Mario Soeiro, Ione Aquemi Guibu, Margô Gomes de Oliveira Karnikowski, Francisco de Assis Acurcio

**Affiliations:** I Programa de Pós-Graduação em Medicamentos e Assistência Farmacêutica. Faculdade de Farmácia. Universidade Federal de Minas Gerais. Belo Horizonte, MG, Brasil; II Departamento de Farmácia Social. Faculdade de Farmácia. Universidade Federal de Minas Gerais. Belo Horizonte, MG, Brasil; III Faculdade de Ciências Médicas. Belo Horizonte, MG, Brasil; IV Instituto de Saúde Coletiva. Universidade Federal da Bahia. Salvador, BA, Brasil; V Departamento de Ciências Farmacêuticas. Universidade Federal de Santa Catarina. Florianópolis, SC, Brasil; VI Núcleo de Estudos de Políticas Públicas. Universidade Estadual de Campinas. Campinas, SP, Brasil; VII Programa de Pós-Graduação em Saúde Coletiva. Departamento de Saúde Coletiva. Faculdade de Ciências Médicas. Universidade Estadual de Campinas. Campinas, SP, Brasil; VIII Programa de Pós-Graduação em Epidemiologia. Faculdade de Medicina. Universidade Federal do Rio Grande do Sul. Porto Alegre, RS, Brasil; IX Faculdade de Ciências Farmacêuticas. Pontifícia Universidade Católica de Campinas. Campinas, SP, Brasil; X Departamento de Saúde Coletiva. Faculdade de Ciências Médicas. Santa Casa de São Paulo. São Paulo, SP, Brasil; XI Faculdade de Ceilândia. Universidade de Brasília. Brasília, DF, Brasil

**Keywords:** Polypharmacy, Risk Factors, Patient Safety, Pharmaceutical Services, Primary Health Care, Health Services Research, Unified Health System, Polimedicação, Fatores de Risco, Segurança do Paciente, Assistência Farmacêutica, Atenção Primária à Saúde, Pesquisa sobre Serviços de Saúde, Sistema Único de Saúde

## Abstract

**OBJECTIVE:**

To characterize the polypharmacy in primary health care patients and to identify its associated factors.

**METHODS:**

This is a cross-sectional, exploratory, and evaluative study, part of the *Pesquisa Nacional sobre Acesso, Utilização e Promoção do Uso Racional de Medicamentos* – *Serviços, 2015* (PNAUM – National Survey on Access, Use and Promotion of Rational Use of Medicines – Services, 2015). The variable of interest was polypharmacy, defined as the use of five or more medicines. We sought to identify the association of sociodemographic variables and indicators of health conditions to polypharmacy. For group comparison, the Pearson’s Chi-square test was used. The association between polypharmacy and explanatory variables was evaluated by logistic regression model (p < 0.05). The quality of the adjustment was verified by Hosmer-Lemeshow test.

**RESULTS:**

The prevalence of polypharmacy among medicine users was 9.4% (95%CI 7.8–12.0) in the general population and 18.1% (95%CI 13.6–22.8) in older adults above 65 years old. We found statistically significant association between polypharmacy and age above 45 years, lower self-perception of health, presence of chronic diseases, having health insurance, care in emergency services, and region of the Country. South users presented the highest chances to polypharmacy. The most used medicines were those of the cardiovascular system, being compatible with the national epidemiological profile.

**CONCLUSIONS:**

Polypharmacy is a reality in the population met within the primary care of Brazilian Unified Health System and may be related to excessive or inappropriate use of medicines. The main challenge to qualify health care is to ensure that prescription of multiple medicines be appropriate and safe.

## RESUMO

**OBJETIVO:**

Caracterizar a polifarmácia em usuários da atenção primária e identificar fatores a ela associados.

**MÉTODOS:**

Estudo transversal, exploratório, de natureza avaliativa, integrante do Componente Serviços da Pesquisa Nacional sobre Acesso, Utilização e Promoção do Uso Racional de Medicamentos, 2015. A variável de interesse foi a polifarmácia, definida como o uso de cinco ou mais medicamentos. Buscou-se identificar a associação de variáveis sociodemográficas e indicadores de condições de saúde à polifarmácia. Para a comparação de grupos utilizou-se o teste qui-quadrado de Pearson. A associação entre polifarmácia e variáveis explicativas foi avaliada por modelo de regressão logística (p < 0,05). A qualidade do ajuste foi verificada pelo teste de Hosmer-Lemeshow.

**RESULTADOS:**

A prevalência de polifarmácia entre os usuários de medicamentos foi de 9,4% (IC95% 7,8–12,0) na população geral e de 18,1% (IC95% 13,6–22,8) em idosos acima de 65 anos. Houve associação estatisticamente significante entre polifarmácia e faixa etária acima de 45 anos, baixa autopercepção de saúde, presença de doenças crônicas, ter plano de saúde, atendimento em serviço de emergência e região do país. Usuários do Sul apresentaram as maiores chances para polifarmácia. Os medicamentos mais utilizados foram os do aparelho cardiovascular, sendo compatível com o perfil epidemiológico nacional.

**CONCLUSÕES:**

A polifarmácia é uma realidade na população atendida no âmbito da atenção primária do Sistema Único de Saúde e pode estar relacionada ao uso exacerbado ou inapropriado de medicamentos. O principal desafio para qualificar a atenção em saúde é garantir que a prescrição de múltiplos medicamentos seja apropriada e segura.

## INTRODUCTION

The provision of health care is quite complex and requires the necessary balance between benefits and risks that accompany the whole process, to provide people the most complete well-being^10^. Patient safety, understood as reduction of the risk of unnecessary damage associated with care, has been considered a priority attribute of the health systems quality throughout the world^10^ ^,^ ^17^. In the context of primary health care, inappropriate correlation between diagnosis and prescribed treatment and misuse of medicines and problems of communication between physicians and patients are the main causes of adverse events^19^.

The use of multiple medications, or polypharmacy, is increasingly common in clinical practice, particularly in people over 65 years old. This growth is related to multiple factors, such as increased life expectancy and the consequent growth in the prevalence of multimorbidity, the enhanced availability of drug treatments and guidelines that recommend the use of more than one drug in the management of several health conditions, such as hypertension and *diabetes mellitus* ^7^ ^,^ ^15^. The optimized association of medicines, prescribed according to the best available evidence, can heal, minimize damage, increase longevity, and improve quality of life^6^. However, some therapies are inadequate and may cause adverse reactions and drug interactions^16^.

One of the challenges in discussing polypharmacy and the associated medication safety is the absence of a universally accepted polypharmacy definition^7^ ^,^ ^21^. Most of the concepts identified by Bushardt et al.^6^ relate the term to exacerbated and inappropriate use of medicines. The reasons that explain this practice are treatments not based on evidence; the adoption of combinations with potential drug interactions; the pharmacological treatment of the side effects of other medicines; and the simultaneous prescription by multiple doctors, without the necessary therapeutic conciliation for the patient.

The inappropriate drug therapy is a serious problem for health systems, being recognized as a costly practice^6^. According to the World Health Organization^28^, about 50% of patients with chronic diseases do not adhere to pharmacological treatments, 4% to 5% of the hospital admissions occur because of preventable adverse events, and about 30% of emergency consultations are generated by medicine-related problems, many of them preventable.

Adverse drug events (ADE) are a common and costly complication in health care, representing the fifth cause of death in the United States^5^. The age of the patient and polypharmacy are factors associated with the higher number of doctor’s appointments and the occurrence of ADE^5^. Avoiding the use of unsuitable and high-risk medicines is an important and effective strategy to reduce the problems related to drug treatment, especially in older adults. However, there are important gaps in the knowledge about this phenomenon, and the best evidence come from hospital environments^28^.

Few studies have assessed polypharmacy in the context of primary health care and in public health systems^28^. Also, the scientific evidence of drug effectiveness come from randomized clinical trials, excluding elderly, people with comorbidities, and polypharmacy^13^. Thus, most of the articles published in the literature do not provide information directly relevant to the people who need drug associations. Studies on these events in the real world are especially important for a better understanding of the challenges in providing quality health care^25^.

The *Pesquisa Nacional sobre Acesso, Utilização e Promoção do Uso Racional de Medicamentos* – *Serviços, 2015* (PNAUM – National Survey on Access, Use and Promotion of Rational Use of Medicines – Services, 2015) aimed to characterize the organization of pharmaceutical services in primary health care of the Brazilian Unified Health System (SUS), to promote access and rational use of medicines, as well as identify and discuss factors that interfere in the consolidation of pharmaceutical services in municipalities. In this context, this study, part of PNAUM – Services, aims to characterize the polypharmacy in patients of the SUS primary health care and to identify its associated factors, to provide subsidies for the improvement of health care in Brazil.

## METHODS

This article is part of PNAUM, a cross-sectional, exploratory, and evaluative study, consisting of an information gathering in a representative sample of cities, primary health care services, patients, physicians, and drug dispensers in the five regions of Brazil. The sampling plan considered the various study populations and estimated the sample sizes for each of these populations^1^. For each region, 120 cities, 300 health services, and 1,800 patients were sampled. The total sample of patients (9,000) was estimated considering the possibility of up to 20% loss. Patients were interviewed in primary health care services, with a specific structured questionnaire. After the training of the interviewers, a pretest was carried out, involving cities with different population sizes to detect opportunities for improving and to validate the questionnaires. The data were collected from July 2014 to May 2015.

Respondents were asked about the knowledge of chronic diseases diagnosed by a health professional; about emergency care and hospitalization in the 12 months preceding the interview; and about physical activity (frequency in the three previous months), smoking (frequency and quantity of cigarettes), and alcohol use (occurrence, frequency, and number of doses). Regarding the use of medicines, all medicines used in 30 days prior to the interview were registered, according to self-report. To ensure greater accuracy, patients were requested to hand in the prescriptions and/or packages of medicines in use, when available. The subjects were divided into three strata, according to the number of medicines used: one, two to four, and five or more medicines. The variable of interest was polypharmacy, defined as the use of five or more medicines. The medicines were classified according to the fifth level of the Anatomical Therapeutic Chemical index (ATC)^29^ and described using the *Denominação Comum Brasileira* (DCB – Brazilian Common Name). The Beers criteria was used to identify the use of potentially inappropriate medications for older adults in the population of the study^3^.

The association between polypharmacy and sociodemographic variables (gender, age, marital status, educational level, economic class, and having health insurance), lifestyle (alcohol consumption, smoking, physical activity practice), and indicators of health conditions (self-perception of health, number and main chronic diseases self-reported, emergency care and hospitalization) was assessed. These variables were included in the study by their epidemiologic importance and for being associated with polypharmacy in several pharmacoepidemiologic studies^4^ ^,^ ^8^ ^,^ ^14^ ^,^ ^21^.

Absolute and relative frequencies were used to describe the variables, using the program R® version 3.2.1, with the *survey* package^a^. For group comparison, the Pearson’s Chi-square test with Rao-Scott correction was adopted, considering a significance level of 5%. The association between polypharmacy and explanatory variables was evaluated by logistic regression model. Univariate models were used to select variables to the multiple model, being eligible those with p-value lower than or equal to 0.20. The selected variables in the univariate models were included in the multiple model. The *backward* method was adopted to obtain the final model, where the variables with p-value lower than 0.05 remained. The adjustment quality was verified by Hosmer-Lemeshow test.

The research was approved by the National Research Ethics Committee (CONEP), under Opinion no. 398,131/2013. All the interviews were preceded by the clarification of the research’s goals to the respondent and by the signature of the informed consent form.

## RESULTS

Interviews were carried out with 8,803 patients in primary health care services of 272 Brazilian cities, 97.8% of the planned sample. Of these, 6,511 used at least one medicine. Polypharmacy (use of five drugs or more) was identified in 9.4% (95%CI 7.8–12.0) of medicine users. The majority of polypharmacy patients were female (79.9%, 95%CI 75.2–83.9), aged between 45 and 64 years (54.8%, 95%CI 49–60.5), married or in common-law marriage (64.6%, 95%CI 58.5–70.3), with some elementary school (54.7%, 95%CI 47.1–62.1%), economic class C (54.0%, 95%CI 48.7–59.3), lived in the South region (49.5% CI95% 34.4–61.0), and without health insurance (83.0%, 95%CI 75.5–88.5). Among those who were on polypharmacy, 32.9% (95%CI 28.2–38.0) were older adults above 65 years old (Table 1). The prevalence of polypharmacy in this group was 18.1% (95%CI 13.6–22.8%).

**Table 1:** Socioeconomic characteristics of medicine users in the SUS primary health care. Nacional Survey on Access, Use and Promotion of Rational Use of Medicines - Services, 2015.

Variable	Number of medicines						p

	1 (n = 2,561)		2–4 (n = 3,340)		5 or more (n = 610)		
		
	n*	% (95%CI)	n*	% (95%CI)	n*	% (95%CI)	
Gender							0.146
Female	1,922	74.7 (71.8–77.5)	2,610	76.7 (74.2–79.0)	492	79.9 (75.2–83.9)	
Male	639	25.3 (22.5–28.2)	730	23.3 (21.0–25.8)	118	20.1 (16.1–24.8)	
Age (years)							< 0.001
18–44	1,541	58.8 (55.9–61.7)	1.266	35.7 (32.2–39.2)	64	12.3 (8.8–17.0)	
45–64	746	30.7 (28.2–33.3)	1.414	43.5 (41.3–45.8)	339	54.8 (49.0–60.5)	
≥ 65	240	10.4 (8.6–12.6)	640	20.8 (18.1–23.9)	207	32.9 (28.2–38.0)	
Marital status							< 0.001
Married/commom-law marriage	1,608	64.2 (61.5–66.9)	2.058	66.4 (64.1–68.7)	350	64.6 (58.5–70.3)	
Single	686	24.5 (22.0–27.2)	718	17.2 (15.5–19.0)	70	9.1 (6.7–12.2)	
Others	267	11.3 (9.6–13.2)	564	16.3 (14.5–18.3)	190	26,3 (21.2–32.1)	
Educational level							< 0.001
Illiterate	187	8.4 (6.7–10.5)	349	11.9 (9.1–15.5)	112	18.5 (13.4–24.9)	
Some elementary or middle school	857	37,9 (34,6–41,3)	1,344	44.7 (40.2–49.2)	309	54.7 (47.1–62.1)	
Elementary or middle school	348	13.7 (10.9–17.2)	416	12.1 (9.9–14.6)	71	10.9 (7.6–15.3)	
High school	964	33.0 (30.1–36.0)	993	25.5 (22.4–28.9)	96	13.3 (9.8–17.8)	
Higher education	205	6.9 (5.5–8.7)	238	5.8 (4.7–7.2)	22	2.6 (1.5–4.4)	
Economic class							0.844
A or B	443	16.0 (13.2–19.2)	532	14.7 (12.2–17.7)	97	14.1 (9.8–19.7)	
C	1,488	54.6 (50.7–58.4)	1,954	54.5 (50.6–58.4)	348	54.0 (48.7–59.3)	
D or F	627	29.5 (24.7–34.7)	853	30.7 (25.6–36.4)	165	31.9 (25.0–39.7)	
Country region							< 0.001
North	491	6.2 (4.7–8.2)	522	4.7 (3.5–6.3)	26	1.2 (0.7–2.3)	
Northeast	483	29.4 (21.5–38.6)	621	29.9 (21.5–39.8)	75	22.1 (13.8–33.5)	
Midwest	507	7.1 (5.3–9.6)	461	4.4 (2.9–6.7)	77	3.5 (1.6–7.5)	
Southeast	571	36.6 (28.3–45.7)	694	32.6 (23.4–43.3)	119	27.2 (18.1–38.9)	
South	494	20.7 (16.2–26.1)	891	28.4 (20.2–38.4)	275	45.9 (34.4–61.0)	
Has health insurance (yes)	215	9.0 (6.6–12.1)	300	9.5 (6.7–13.3)	89	17.0 (11.5–24.5)	0.001

* non-weighted n-value.

Source: PNAUM- Services, 2015.

Regarding the self-reported lifestyle and health characteristics, 5.9% (95%CI 3.8–9.1) of patients on polypharmacy consumed alcohol more than once per month, 14.7% (95%CI 10.6–20.1) were smokers, and 29.6% (95%CI 24.7–35.0) practiced physical exercise or sports in the three months preceding the interview (Table 2). Most polypharmacy patients classified their health as neither bad/nor good (51.1%, 95%CI 45.3–56.9) and reported having two or more chronic conditions (95.1%, 95%CI 92.1–97.0). The main reported diseases were hypertension (84.3%, 95%CI 79.9–87.9), dyslipidemia (57.8%, 95%CI 51.8–63.6), arthritis, arthrosis, or rheumatism (51.3%, 95%CI 44.8–57.7), and depression (47.3%, 95%CI 40.9–53.9), and *diabetes mellitus* (41.6%, 95%CI 36.6–46.8). Among the 25 most used medicines by polypharmacy patients, 13 (52.0%) belong to group C of the ATC, having action on the cardiovascular system. The most used drugs were simvastatin, losartan, and omeprazole (Table 3). Among the most used medicines, we highlight amitriptyline, clonazepam, diazepam, fluoxetine, and ibuprofen, belonging to the list of potentially inappropriate items for use in older adults, according to Beers criteria^3^.

**Table 2:** Lifestyle characteristics and health condition indicators of patients attended by SUS primary health care. National Survey on Access, Use and Promotion of Rational Use of Medicines – Services, 2015.

Variable	Number of Medicines						p

	1 (n = 2,561)		2–4 (n = 3,340)		5 or more (n = 610)		
		
	n^a^	% (95%CI)	n^a^	% (95%CI)	n^a^	% (95%CI)	
Alcohol use (yes)^b^	339	12.9 (11.0–15.1)	343	9.4 (7.9–11.1)	40	5.9 (3.8–9.1)	< 0.001
Smoking (yes)	319	13.2 (11.6–15.0)	405	13.4 (11.5–15.5)	87	14.7 (10.6–20.1)	0.783
Practice of physical activity (yes)^c^	662	23.6 (20.5–27.0)	924	27.9 (24.0–32.2)	184	29.6 (24.7–35.0)	0.007
Self-perception of health							< 0.001
Very good/good	1,645	63.0 (59.2–66.5)	1,613	48.5 (44.8–52.2)	173	25.7 (21.8–30.2)	
Neither bad/nor good	777	31.7 (29.2–34.3)	1,388	41.9 (39.0–44.9)	307	51.1 (45.3–56.9)	
Bad/very bad	135	5.4 (3.8–7.5)	334	9.6 (8.1–11.3)	129	23.2 (18.5–28.6)	
Number of chronic diseases							< 0.001
None	1,036	39.0 (36.0–42.2)	550	24.8 (22.1–27.7)	8	1.0 (0.4–2.5)	
One	840	36.5 (33.4–39.7)	792	24.8 (22.1–27.7)	30	27.2 (25.5–29.1)	
Two or more	595	24.5 (22.1–27.0)	1,846	60.6 (55.9–65.0)	532	95.1 (92.1–97.0)	
Main chronic diseases							< 0.001
Hypertension	642	28.1 (25.1–31.4)	1,787	56.6 (51.6–61.4)	510	84.3 (79.9–87.9)	
Dyslipidemia	367	14.4 (12.5–16.6)	1,036	31.6 (28.5–34.9)	338	57.8 (51.8–63.6)	
Arthritis, arthrosis, or rheumatism	293	12.4 (10.4–14.6)	896	26.5 (23.2–30.1)	306	51.3 (44.8–57.7)	
Depression	281	12.2 (10.5–14.2)	784	24.5 (21.5–27.9)	277	47.3 (40.9–53.9)	
*Diabetes mellitus*	154	5.4 (4.5–6.5)	653	21.1 (17.6–25.0)	270	41.6 (36.6–46.8)	
Heart diseases	99	3.4 (2.4–4.7)	300	9.9 (8.1–12.1)	198	33.3 (27.0–40.3)	
Chronic pulmonary disease	210	8.4 (6.9–10.1)	401	10.7 (9.0–12.6)	121	18.5 (14.4–23.5)	
Stroke	29	1.4 (0.8–2.2)	111	3.3 (2.4–4.4)	60	8.7 (6.2–12.1)	
Other diseases	396	15.1 (12.5–18.2)	760	23.7 (19.7–28.3)	223	38.7 (31.8–46.2)	
Emergency care (yes)^d^	550	19.3 (16.8–22.1)	1,004	26.1 (22.9–29.6)	236	39.4 (34.0–45.2)	< 0.001
Hospitalization (yes)^d^	203	7.9 (6.5–9.5)	383	10.8 (8.8–13.2)	113	19.9 (15.4–25.3)	< 0.001

^a^ non-weighted n-value.

^b^ Alcohol use considered positive for report of more than once per month.

^c^ Report of practice of physical activity or sport in the three months preceding the interview.

^d^ Self-report referring to the 12 months prior to the interview.

Source: PNAUM – Services, 2015.

**Table 3:** Most used medicines by polypharmacy patientsa of primary health care services, according the *Anatomical Therapeutic Chemical Classification Index*. National Survey on Access, Use and Promotion of Rational Use of Medicines – Services, 2015.

Medicines	ATC Code (5^th^ level)^b^	n^c^	% (95%CI)
Simvastatin	C10AA01	224	35.7 (29.9–42.0)
Losartan	C09CA01	213	34.0 (26.9–41.8)
Omeprazole	A02BC01	200	33.6 (28.3–39.4)
Acetylsalicylic acid	N02BA01	175	26.5 (20.3–33.7)
Metformin	A10BA02	161	24.8 (18.1–33.0)
Hydrochlorothiazide	C03AA03	159	23.5 (16.8–31.9)
Enalapril	C09AA02	101	15.8 (10.9–22.3)
Atenolol	C07AB03	101	15.0 (10.3–21.5)
Captopril+diuretic	C09BA01	65	12.2 (7.0–20.5)
Fluoxetine^d^	N06AB03	64	12.2 (9.3–15.9)
Glibenclamide	A10BB01	67	11.4 (7.7–16.5)
Captopril	C09AA01	61	11.3 (8.0–15.7)
Clonazepam^d^	N03AE01	60	11.2 (8.9–13.9)
Dipyrone	N02BB02	50	10.0 (6.5–15.2)
Ibuprofen^d^	C01EB16	52	9.7 (7.1–13.2)
Propranolol	C07AA05	52	8.8 (6.2–12.5)
Paracetamol	N02BE01	58	8.7 (6.6–11.3)
Furosemide	C03CA01	55	8.7 (6.8–11.0)
Amlodipine	C08CA01	64	8.5 (5.6–12.8)
Losartan+diuretic	C09DA01	41	8.3 (4.0–16.3)
Diazepam^d^	N05BA01	40	7.4 (3.9–13.6)
Diclofenac	M01AB05	30	6.8 (4.6–10.1)
Amitriptyline^d^	N06AA09	48	6.6 (4.5–9.7)
Metformin+sulfonylurea	A10BD02	24	6.0 (2.0–17.1)
Atenolol+thiazides	C07BB03	33	5.3 (2.4–11.5)

^a^ Use of five medicines or more.

^b^ Classification according to the WHO Collaborating Centre for Drug Statistics Methodology – Anatomical Therapeutic Chemical (ATC) Classification Index 2016.

^c^ non-weighted n-value.

^d^ Potentially inappropriate medicines for use in people over 65 years old, according to Beers criteria.

In Fick et al., 2003.

Source: PNAUM – Services, 2015.

The results of univariate and multiple logistic models for the polypharmacy predictors are presented in Table 4. Individuals with health insurance were 1.6 times more likely to be in polypharmacy than those without it. Polypharmacy was significantly associated with age, being higher in older adults over 65 years old (OR 1.95 for people aged between 45 and 64 years old and OR 2.43 for the age group of 65 years old or more). There was association between polypharmacy and self-perception of health, and this association was inversely proportional to the worsening of self-perception (neither bad nor good OR 1.82; 95%CI 1.40–2.38; bad/very bad OR 2.91; 95%CI 1.93–4.38), to the report of having emergency health care (OR 1.59; 95%CI 1.19–2.11) and to having chronic diseases, with the highest association in hypertensive patients (OR 3.49; 95%CI 2.43–5.21). The association between the Country regions and polypharmacy was variable, and living in the South region showed higher association (5.8 times higher than in the North).

**Table 4:** Associated factors (odds ratio) with polypharmacy among patients of SUS primary health care services. National Survey on Access, Use and Promotion of Rational Use of Medicines – Services, 2015.

Variable	Univariate		p	Multiple^a^		p
	
	OR	95%CI		OR	95%CI	
Intercept	–	–	–	0.004	0.002–0.008	< 0.001
Sex						
Male	–	–	–			
Female	1.266	0.954–1.680	0.103			
Age (years)						
18–44	–	–	–	–	–	–
45–64	5.279	3.499–7.964	< 0.001	1.946	1.275–2.970	< 0.001
≥ 65	7.316	4.904–10.914	< 0.001	2.428	1.541–3.825	< 0.001
Education level						
Higher education	–	–	–			
High school	1.131	0.603–2.121	0.702			
Elementary or middle school	2.069	1.051–4.071	0.036			
Some elementary or middle school	3.176	1.768–5.706	< 0.001			
Illiterate	4.296	2.177–8.474	< 0.001			
Marital status						
Single	–	–	–			
Married/common-law marriage	2.202	1.572–3.084	< 0.001			
Others	4.119	2.804–6.049	< 0.001			
Economic class						
A or B	–	–	–			
C	1.072	0.764–1.504	0.686			
D or E	1.143	0.717–1.823	0.574			
Has health insurance						
No	–	–	–	–	–	–
Yes	2.004	1.582–2.538	< 0.001	1.602	1.125–2.278	0.009
Hypertension						
No	–	–	–	–	–	–
Yes	6.633	4.765–9.234	< 0.001	3.496	2.345–5.211	< 0.001
*Diabetes mellitus*						
No	–	–	–	–	–	–
Yes	4.177	3.226–5.408	< 0.001	2.297	1.753–3.009	< 0.001
Depression						
No	–	–	–	–	–	–
Yes	3.725	3.002–4.623	< 0.001	2.381	1.862–3.044	< 0.001
Arthritis						
No	–	–	–	–	–	–
Yes	4.052	3.179–5.163	< 0.001	1.736	1.314–2.295	< 0.001
Self-perception of health						
Good/Very good	–	–	–	–	–	–
Neither good nor bad	2.871	2.289–3.602	< 0.001	1.823	1.400–2.375	< 0.001
Bad/Very bad	6.244	4.525–8.615	< 0.001	2.912	1.935–4.380	< 0.001
Country region						
North	–	–	–	–	–	–
Midwest	2.820	1.359–5.851	0.005	2.304	1.096–4.844	0.027
Northeast	3.542	1.814–6.913	< 0.001	2.767	1.358–5.638	0.005
Southeast	3.648	1.894–7.024	< 0.001	2.621	1.266–5.430	0.010
South	8.829	4.867–16.015	< 0.001	5.815	3.133–10.794	< 0.001
Emergency care						
No	–	–	–	–	–	–
Yes	2.144	1.688–2.722	< 0.001	1.592	1.198–2.114	< 0.001
Hospitalization						
No	–	–	–	–	–	–
Yes	2.346	1.718–3.204	< 0.001			

^a^ Hosmer-Lemeshow test = 0.4228.

Source: PNAUM – Services, 2015.

## DISCUSSION

The prevalence of polypharmacy observed in this study (9.4%, 95%CI 7.8–12.0) was similar to that of primary health care in Germany (10.0%)^12^ and lower than the 20.8% in adults attended by primary health care in Scotland^13^. Polypharmacy in people over 65 years old (18.1%) was higher than the 11.0% in older adults from areas covered by the *Estratégia Saúde da Família* (ESF – Family Health Strategy) in Recife, state of Pernambuco^21^, but lower than that found in other studies: 28.0% in the older adults in Goiânia^27^, 32.7% among retirees in Rio de Janeiro^24^, 36% in the older adults in the city of São Paulo^8^, and 35.8% in North-American older adults^22^.

Studies on polypharmacy in primary health care, including the general population, are scarce^12^ ^,^ ^13^ ^,^ ^19^. Considering the process of population aging and evidence about the relationship between increasing age and number of prescription drugs, professionals must ensure the quality of pharmacotherapy in the health care process, avoiding the excessive use of multiple medicines^7^ ^,^ ^13^ ^,^ ^15^. Polypharmacy has been linked to negative health outcomes; increased morbidity and mortality; reduction of the quality of life, especially in older adults; and increased costs of care, with impact to patients and health care systems^7^.

In recent years, geriatric polypharmacy has significantly increased. This group typically has a high comorbidity index, high risk for prescription of potentially inappropriate medicines, and are more susceptible to loss of doses or administration errors, which may impair adherence to treatment^7^ ^,^ ^20^. In addition, they often present a compromised nutritional status and pharmacokinetic and pharmacodynamic changes inherent to the aging process. These characteristics explain the greater vulnerability of older adults to the occurrence of adverse events, reduction of therapeutic efficacy, and increased risk of drug interactions^8^ ^,^ ^20^ ^,^ ^21^ ^,^ ^27^. Observational studies have shown a strong relationship between the use of potentially inappropriate medications and adverse health outcomes, such as ADE (gastrointestinal bleeding, sedation, delirium, falls and fractures), hospitalization, and death^2^.

Although most studies investigated polypharmacy in older adults, this study showed a significant association between the age group of 45 to 64 years old and the use of five or more drugs. These data need to be better understood to guide public policies and qualify the assistance in primary health care. The model “one disease – one drug therapy” is an inadequate approach in face of the epidemic of medicine use in the 21^st^ century. As pointed out by Bjerrum et al.^4^, the implementation of information strategies for general practitioners on prescribing in primary care can improve this practice and reduce polypharmacy, qualifying health care.

The variables gender, economic class, marital status, and education level were less relevant in determining the consumption of multiple medicines in SUS primary health care. Loyola Filho, Uchoa, and Lima-Costa^14^ highlighted that only isolated studies have identified a relationship between higher education level, widowhood, and polypharmacy. Regarding sex, most national and international surveys^4^ ^,^ ^8^ ^,^ ^9^ ^,^ ^14^ ^,^ ^27^ indicate that women seek more health services and that conditions inherent to their reproductive role, such as pregnancy and contraception, may explain the increased use of medicines^14^ ^,^ ^15^. However, this study did not observe association between gender and polypharmacy. Consistent with this result is the study of O’Dwyer et al.^22^ in a population over 40 years old in Ireland, which also found no association between polypharmacy and female sex.

Similar to other national studies^8^ ^,^ ^21^, individuals with health insurance presented a higher chance of polypharmacy. They have greater access to appointments with medical specialists, expanding the variety of prescriptions^2^ ^,^ ^8^. According to Neves et al.^21^, general practitioners of the Family Health Strategy normally prescribe items from SUS essential list, to enable the free drug supply. This practice reduces the amplitude of the therapeutic arsenal and, consequently, the average number of medicines prescribed per patient.

The self-assessment of health as regular and bad/very bad, observed by other studies^8^ ^,^ ^14^ ^,^ ^15^ ^,^ ^21^, showed positive association with polypharmacy. According to Carvalho et al.^8^, these findings are consistent because the connection between health problems and medicine use is obvious. These results are reinforced by the association also verified with the emergency medical service, which may be related to a worse health condition. Furthermore, Mira et al.^19^ identified that 5% of medication errors in polypharmacy patients had serious consequences, requiring emergency care or hospitalization.

The association between self-report of hypertension, *diabetes mellitus*, depression, rheumatic diseases, and polypharmacy is in line with other national and international studies^4^ ^,^ ^8^ ^,^ ^15^ ^,^ ^24^. These are prevalent conditions in the Country, especially in older adults, whose control and treatment require the use of medicines. There is consistency between the 25 most used medicines by polypharmacy individuals and self-reported diseases. Hypertension, corroborating other studies^8^ ^,^ ^21^, was the most frequent chronic disease and presented the most intense association with polypharmacy.

The higher prevalence of medicines for the cardiovascular system (ATC group C) in the polypharmacy population corroborates with Charlesworth et al.^9^, who observed a relationship between the classes of antihypertensives, statins, and antidiabetics and the increase in the prevalence of drug use in American older adults. Qato et al.^23^ observed a statistically significant increase in the prescription of statins in the United States of America, reaching 46.2% of people over 65 years old, in 2011. Evidence on the clinical use of statins are controversial, with some studies showing its usefulness in reducing morbidity and mortality and others showing its potential damage^11^.

The high use of omeprazole in this study may be explained, according to Carvalho et al.^8^, by the prophylactic and not always rational prescription of drugs for reducing gastric acidity. Often, an adverse reaction can be interpreted as new clinical entity, being treated with new medication, which constitutes an iatrogenic cascade^8^. Bjerrum et al.^4^ point out that some authors consider polypharmacy an “uncontrolled experiment,” because most individuals has a unique combination of medicines and, therefore, require individualized care and therapeutic conciliation.

Among the most used medicines by the polypharmacy patients, we highlight five items (amitriptyline, clonazepam, diazepam, fluoxetine, and ibuprofen), which belong to the list of potentially inappropriate medications for use in older adults, according to Beers criteria^3^. These results are very relevant when considering that the age group above 65 years old was associated with the greatest chance of polypharmacy within the primary health care of SUS. The Beers criteria is an important measure of quality of health care in older adults, and it should be incorporated into electronic record systems to support the prescription process and to identify situations in which non-pharmacological alternatives would be more appropriated^2^ ^,^ ^3^.

The polypharmacy varied according to Brazilian regions, with greater association in the South and Southeast. These results can be explained by the characteristics of the population sampled, because individuals of these regions showed higher prevalence of comorbidities. In addition, the states of those regions have a higher health care insurance coverage, variable predictive of polypharmacy in this study^18^.

As Rozenfeld et al.^24^ point out, polypharmacy is not always a preventable event. Chronic diseases of high prevalence, such as hypertension and *diabetes mellitus*, are typically handled by association of medicines. The review of drugs and the potential deprescription should be evaluated mainly by physicians or pharmacists, to customize the treatment in people with multimorbidity or specific vulnerability. Also, the monitoring for the potential occurrence of drug interactions is important^13^. According to Secoli et al.^26^, the evaluation of multiple therapeutic regimens, especially in older adults, allows the identification of associations with potential drug interactions and their suspension, to minimize the damage and qualify the medicine use. Protocols and guidelines for the management of the most prevalent chronic diseases must contain, in addition to the treatment indications, recommendations about situations where deprescription can be adopted.

There is evidence that prescribers’ education, with emphasis on stimulating preventive practices, impacts adherence to therapy and the quality of medicines use^25^. Mira et al.^19^ found that only 32.5% of patients attended by the Spanish primary health care were asked by physicians about drugs prescribed by other health professionals. In this study, the medication errors were associated with: feeling of not being listened to, loss of confidence in the physician, occurrence of simultaneous prescriptions by several medical specialists, and incongruent information between different health professionals.

Pharmaceutical care services, with appointments to develop care plans, to solve problems related to medicines, and to provide timely follow-up, focusing on the acquisition of skills for the co-responsibility, can improve adherence to drug therapy and, consequently, clinical outcomes. The adoption of strategies for the self-registration of all medicines used by patients, including medicinal plants, non-prescription drugs, and dietary supplements, may contribute to the clinical history improvement, reducing the memory bias^6^.

This study has some limitations. Because it is the first nationwide study on polypharmacy in patients of public health services, there are methodological differences and diversity between the populations of the studies used to data discussion, which restricts the direct comparison. Another limitation refers to the polypharmacy concept adopted, considered only as the concomitant use of multiple medicines. We did not verify the reasons for drug prescription, to assess the appropriateness of the use of each medicine. In addition, the data presented may be underestimated by memory constraints, because medicines and diseases were self-reported by patients. Because it is a cross-sectional study, it is not possible to establish the temporality of the associated factors.

Despite the presented limitations, the results showed a high rate of polypharmacy, especially in the elderly population, which needs to be better understood by managers and multidisciplinary health teams. Reducing avoidable complications and medication errors are increasing needs and should promote the adoption of safe practices, based on scientific evidence. According to Bjerrum et al.^4^, programs designed to reduce problems associated with polypharmacy are more effective when developed for subgroups of patients with increased risk. In the health care process, the establishment of co-responsibility and solidarity links with patients and their families contribute to the strengthening of the patient’s safety^17^.

In conclusion, polypharmacy is a reality in the population attended by the SUS primary health care. The recent epidemiological changes, with the increase in life expectancy and, consequently, chronic diseases, has changed the perspective on the use of multiple medicines in health care. Considering the important role of the drug public supply in Brazil, this study provides subsidies for the improvement of prescribing practices and use of medicines. Activities to increase drug safety in sub-populations with a greater chance of polypharmacy have the potential to reduce preventable adverse events, especially in older adults.

The number of drugs prescribed should consider the real needs of each patient and the balance between potential benefits and risks. The main challenge to qualify health care is to ensure that the prescription of multiple drugs is appropriate and safe. Regular evaluation of the therapeutic regimens, focusing on adherence, adjustment to individual preferences, and risk identification can minimize the damage and maximize the benefits. Continued professional training, multidisciplinary team work, and population education are important strategies to qualify the use of medicines and strengthen the *Política Nacional de Segurança do Paciente* (PNSP – National Policy for Patient Safety).

## Footnotes

a T. Lumley “survey: analysis of complex survey samples”. R package version 3.30. Vienna: The R Foundation; 2014.
